# Nursing Home Admission Decisions During the COVID-19 Pandemic: A Resource Dependence Perspective

**DOI:** 10.1093/geroni/igaf122.2309

**Published:** 2025-12-31

**Authors:** Zihan Chen, Talia Benheim, Courtney Hawes, Joan Brazier, Amy Meehan, Emily Gadbois

**Affiliations:** Brown University, Providence, Rhode Island, United States; Brown University, Providence, Rhode Island, United States; Brown University, Providence, Rhode Island, United States; Brown University, Providence, Rhode Island, United States; Brown University, Providence, Rhode Island, United States; Brown University School of Public Health, Providence, Rhode Island, United States

## Abstract

During the COVID-19 pandemic, hospitals needed to efficiently discharge stabilized patients who still required care. Although nursing homes were potential care settings, most facilities did not admit COVID-19 patients during the pandemic. This study used qualitative methods to examine environmental factors that shaped nursing home admission strategies for COVID-19 patients. We conducted 156 in-depth, semi-structured qualitative interviews with administrators from 40 nursing homes in eight U.S. healthcare markets. Each participant participated in four interviews between July 2020 and December 2021. The research team created and iteratively refined a coding scheme and applied that scheme to the transcripts of audio recordings. Using Resource Dependence Theory, we identified key themes related to nursing home environments, relationships with external stakeholders, and responses to market uncertainties. First, the pandemic led to decreased concentration in regulatory authority, and to shortages in critical resources, such as staffing and physical capacity. Second, nursing homes navigated competing pressures from key actors, including hospitals, regulators, patients’ families, and the broader community. Some urged facilities to admit COVID-19 patients, while others strongly opposed it. Third, to manage market uncertainties and conflicting demands, many nursing homes implemented new admission strategies, such as halting admissions early in the pandemic, or forming collaborations with other nursing facilities to coordinate patient referrals and discharges. Our findings highlight policies to improve care for vulnerable populations in future pandemics, such as disseminating consistent regulations and supporting communication channels among providers.

